# Effects of COVID-19 on University Student Food Security

**DOI:** 10.3390/nu13061932

**Published:** 2021-06-04

**Authors:** Elizabeth D. Davitt, Michelle M. Heer, Donna M. Winham, Simon T. Knoblauch, Mack C. Shelley

**Affiliations:** 1 Department of Food Science & Human Nutrition, Iowa State University, Ames, IA 50010, USA; eddavitt23@gmail.com (E.D.D.); mmheer@gmail.com (M.M.H.); simonk@iastate.edu (S.T.K.); 2 Departments of Political Science and Statistics, Iowa State University, Ames, IA 50010, USA; mshelley@iastate.edu

**Keywords:** college students, food insecurity, coronavirus, young adults

## Abstract

During COVID-19 restrictions in spring 2020, college students experienced closed dormitories and increased unemployment and many students moved in with their families. College students were vulnerable to food insecurity pre-pandemic and this study examined how the living situations and food security status changed for Midwestern university students due to COVID-19 restrictions. An email survey administered to Iowa State University students between the ages of 18 and 30 who physically attended campus prior to its closure produced 1434 responses. Students living with a parent or guardian increased by 44% and were less likely to experience food insecurity or less likely to work. They had lower stress and ate more home-cooked meals. Students living on their own had higher rates of food insecurity, greater stress, poorer health status, higher cooking self-efficacy, and worked more hours. Seventeen percent of all students were food insecure; related factors were non-White ethnicity, lower cooking self-efficacy, undergraduate status, receipt of financial aid, employment, stress, living in the same situation as before the campus closure, and consumption of more take-out or fast food. These individuals had more barriers to food access. Knowledge of these factors provide useful information to inform future support services for this population in similar conditions.

## 1. Introduction

The economic impacts of the coronavirus disease 2019 (COVID-19) pandemic included increased unemployment, altered social structures, upset housing arrangements, and decreased food security in the United States (US) [1,2,3]. The US saw a distinct rise in food insecurity due to this unprecedented global crisis [4]. Between May and July 2020, 22.5% of people nationally and 18.1% of people in Iowa reported experiencing food insecurity [5] compared to 10.5% of US households in 2019 [6].

The COVID-19 pandemic uniquely affected college students due to the closure of campus dormitories and limited campus dining and food outlet availability for the latter part of the spring 2020 semester [7]. Across the US, 1100 colleges and universities transitioned to the online class format for virtual instruction [8] in March 2020. Many students returned home to live with their parents or families because of campus shutdowns, although travel restrictions left some international students unable to return to their native country [7].

Individuals are classified as food insecure if they report diminished variety, quality, and desirability of a diet as well as decreased access to food [9]. College students experienced a higher degree of food insecurity than the general population prior to the pandemic [10]. In addition to financial limitations, college students may be more susceptible to food insecurity due to housing uncertainty, higher perceived stress, and lower awareness and/or use of public benefits [10]. A 2019 national survey found that approximately 41% of students at 68 four-year institutions were food insecure [10]. Previous 2018 and 2019 research with students at Iowa State University (ISU) in the Midwest US found that 24% to 28% were food insecure [11,12]. Food-insecure students are more likely to report eating less, being hungry, lacking balanced meals, and having poorer health [13]. Beyond the immediate concern for hunger, food insecurity can predict increased risk for chronic disease later in life due to poor nutrition [14].

Nationally, unemployment rates for those between 18- and 24-year-old during the COVID-19 pandemic were among the highest by age category and this peaked during April and May 2020 [15]. Emergent pandemic circumstances disproportionally affected people who were already food insecure and pushed many more into new food crises [4]. The direct effect of these changes on college students was unknown but pandemic-related closures of campuses and businesses displaced student workers.

At the beginning of the pandemic, the US saw a change in consumer behavior and an influx of panic buying of certain grocery items [2]. For the week ending 7 March 2020, there were sales increases in shelf-stable proteins (dried beans +63%, chickpeas +47%, black beans +41%, canned meat +58%, and tuna +31%) and staples, such as rice (+57%) and water (+42%) [16]. Whether or not college students similarly modified their purchasing behavior to accommodate the pandemic changes remains to be determined.

As college students are vulnerable to food insecurity, this study sought to increase the understanding of how that risk changes during a crisis such as the COVID-19 pandemic [17]. Specifically, this cross-sectional descriptive study describes the COVID-19-related behavioral, dietary, and food purchasing practices of ISU students approximately six weeks after campus closure and shift to online learning. The researchers hypothesized that employment and living situation changes related to the pandemic were associated with food insecurity among students at ISU. The objectives for this study were to (1) identify how the shift to online instruction and campus shutdown affected student behaviors, living situations, and food procurement and preparation practices; (2) to describe and compare the demographic and socioeconomic factors of students by food secure or insecure status; and (3) describe the risk factors and co-occurring factors experienced by food-insecure college students under COVID-19 conditions. This research work will provide useful information to inform support services that may need to address this issue in the future.

## 2. Materials and Methods

### 2.1. Study Design and Participants

Students aged 18–30 years enrolled at ISU as of March 2020 and that were physically present in the campus area prior to closure were eligible to complete an online survey on food security and changes in food practices that resulted from the pandemic. Due to COVID-19 precautions, students were asked not to return to campus after dismissal for spring break (16–20 March 2020). Between 26 and 30 April 2020, one email invitation was sent to 29,810 students’ university email addresses via Survey Monkey software (San Mateo, CA, USA). Professional students in the College of Veterinary Medicine were ineligible and not invited. Those studying abroad or those exclusively enrolled in online courses and otherwise off-site (prior to the virtual learning mandate) were ineligible. Of the invitees, 19,152 opened the email, 1907 started the survey, and 1434 had complete data for analysis (7.5% response rate). No reminder emails were sent due to the limited funding for incentives and the large pool of participants (*n* = 1434). Information on informed consent, including the possibility of deidentified data sharing, was on the first page of the survey. Completion of the survey was considered informed consent. The average time spent on the survey was 17 min. A $5 gift card to a major retailer was provided to respondents who answered at least 75% of the survey. The Iowa State University Institutional Review Board approved the study protocol (#16-289). Data from this study on pulse (dry beans, peas, lentils, and chickpeas) intakes and knowledge, and predictors of plant-based alternatives to meat consumption are reported elsewhere [18,19].

### 2.2. Survey Development

Demographic, housing, and employment questions were adapted from two validated college student surveys [20,21]. Questions on the frequency of types of meals consumed (homemade and fast food, etc.) and those related to COVID-19 lifestyle changes were included [22,23]. Questions were adapted to evaluate types of food items purchased and stocked up on [16]. A validated ten-item food frequency screener for fruits, vegetables, and fiber was used to provide estimates of dietary intake [24,25]. Food security during the last 30 days was measured using the USDA ERS six-item Core Food Security Survey Module [26]. Seven cooking self-efficacy questions were asked to assess confidence in meal preparation skills (not at all, a little, somewhat, and very confident) [27]. Two integrity checks for the seriousness and sincerity of responses were built into the survey (level of honesty and the degree of accuracy in answers) [28].

### 2.3. Data Transformations and Analysis

Data were downloaded from Survey Monkey into SPSS v. 26 (IBM, Armonk, NY, USA) for analysis. After examination for normality of the frequency distributions, some categories with less than 5% of responses were combined to form approximately equal tertiles or quartiles, e.g., self-reported health, alcohol consumption, hours worked, and housing locations. The food security total scores were categorized into high, low, and very low per instrument guidelines [26]. The cooking self-efficacy questions were totaled to create a score on a scale from 4 to 28. Comparisons by bivariate residence type (with parents/on own) and food security status (secure/insecure) were conducted using chi-square for independence and analysis of variance (ANOVA). Those factors that demonstrated statistically significant differences by food security status or that were theoretically important (e.g., gender) were entered into logistic regression analysis to predict food insecurity. A *p*-value < 0.05 was considered statistically significant.

## 3. Results

Of the 1907 total respondents, 164 were ineligible and 271 had incomplete data. Thirty-eight participants failed the integrity checks that gauged the accuracy of responses. The demographic characteristics of the remaining 1434 participants are displayed in Table 1. The analysis sample was 61% female, 82% non-Hispanic White, 82% undergraduates, and 83% single, with mean age of 21.4 ± 2.7 years. Fifty-six percent of respondents were from Iowa, 35% were from other US states, and 9% were from outside the US. University enrollment data from spring 2020 indicated that the overall population was 43% female, 85% White, 60% from Iowa, 35% from other US states, and 5% from outside the US [29].

Slightly more than half (53%) of students reported to be living at home with parents/guardians and 47% reported to be living on their own (alone or with roommates or a partner). Significantly more students who were younger, female, White, undergraduate, from the US, and single reported they were living at home. Overall, 17% of students surveyed reported experiencing food insecurity. Significantly more food-insecure students were living on their own, were non-White, international, and married in comparison to those categorized as food secure. A higher number of food-insecure students received financial aid, had higher BMIs, and self-reported poorer health status (Table 1).

### 3.1. COVID-19-Related Behaviors

COVID-19-related behaviors are shown in Table 2. Regarding housing, 50.8% of participants did not change residence and 49.2% moved housing locations in March 2020. Notably, almost 31% were required to move due to COVID-19 policy changes alone. The proportion of students who lived with a parent or guardian increased from 9% pre-COVID-19 to 53% at the time of the survey and the proportion who lived in on-campus housing decreased markedly with COVID-19 restrictions.

Students who returned home were more likely to report that they were not working for pay during the semester or had lost their job due to the pandemic shutdown. Those living on their own were more likely to be working 11 hours or more per week. Students living at home with family members were significantly more likely to self-isolate than students living on their own. Fifty-four percent of students reported more than average or tremendous stress, with significantly higher rates of stress in students living on their own. Students living at home were more likely not to drink alcohol at all, while those living on their own were more likely to binge drink one or more times in the last month. Dietary intakes of fruits and vegetables were significantly lower for students living on their own (Table 1; also see Supplementary Table S1 for frequencies of the 10 food items from the food frequency screener).

There were significant differences in housing and COVID-19-related behaviors by food security status. Fewer food-insecure individuals moved and one-quarter lost their jobs, most likely due to the shutdown in March 2020. Food-secure students were more likely to work less than ten hours per week or not at all. Food-secure students reported average stress more often, while food-insecure students were more likely to say they experienced tremendous stress. Food-insecure students were more likely to binge drink four or more times during the previous month. Although there were no significant differences in total daily fruit and vegetable consumption based on food security status, food-insecure students were less likely to consume whole fruit and “other vegetables.” There were no significant differences between cohorts for other categories including fibers, such as cereals and dark breads; and beans, such as peas and lentils (Table S1).

### 3.2. Food Acquisition, Preparation, Cooking Self-Efficacy, and Barriers to Access

Food acquisition, preparation, and barriers to food access are shown in Table 3. Regarding living circumstances, few students living at home bought their own food or prepared it. Household shopping frequency was higher for those at home than for students living independently. Barriers to food access were significantly greater for students living on their own for all nine questions. Significantly more students living on their own used food assistance resources. They also had significantly higher cooking self-efficacy (Table 3; also see Supplementary Table S2 for individual components of cooking self-efficacy evaluation). Students living on their own were significantly more confident when it came to preparing meals, such as a stir-fry, casserole, or one from scratch without a recipe.

Regarding food security status, over half of the food-insecure students were the main food shopper and meal preparer in the household and they shopped significantly less frequently compared to food-secure respondents. Barriers to food access were significantly greater for food-insecure students than their food secure peers. Significantly more food-insecure students utilized food assistance programs. The average cooking self-efficacy score was significantly higher for food-secure students. They were significantly more confident about five of the seven items then the food-insecure students (Table S2).

### 3.3. Meal Source and Stocking Up on Food Products

Meal source and purchasing habits of shelf-stable foods during the early stages of the pandemic are shown in Table 4. Questions were asked regarding behaviors in the past 30 days. Overall, most students consumed homemade meals (85%) three or more days per week. Students living at home ate homemade meals significantly more frequently and students living on their own reported significantly higher consumption of fast food on a weekly basis. Food-secure students consumed homemade food significantly more frequently, while their food-insecure peers significantly more often reported consuming microwave or frozen meals, take-out food, and fast food three or more days per week.

Overall, the most popular food items to stock up on were pasta (23%), rice or potatoes (19%), and canned or frozen fruits and vegetables (17% and 16%, respectively). The most routinely purchased items were rice or potatoes (68%), snacks (65%), and pasta (62%) while over 60% of students did not buy dry beans, lentils, chickpeas, or canned meats.

A significantly greater percentage of students living at home reported purchasing foods from all categories routinely compared to those living on their own. Students living at home stocked up on canned fruits and vegetables more frequently and students living on their own reported more first-time purchases of snack foods. Food-secure students routinely bought food from all categories with the exception of canned meat and dry beans, lentils, and chickpeas. Food-insecure students more frequently indicated they had either made a first-time purchase or did not buy pasta, canned beans or legumes, frozen fruit and vegetables, and snacks. They were also more likely to report stocking up on rice or potatoes, snack foods, and pulses (dry beans, lentils, and chickpeas). Rice or potatoes and canned fruits and vegetables were cited as first-time purchases among food-insecure students.

### 3.4. Predictive Variables of Food Insecurity

In order to document the demographic and socioeconomic variables affecting student food security, multiple predictors—cooking self-efficacy mean score, living with parents or on their own, undergraduate status, race (White or other), stress level, employment status, loss of job due to COVID-19, and currently working—were entered into a logistic regression model to predict food security status (food secure and food insecure) (Table 5). The model correctly predicts 84% of food security status overall, including 99% of food-secure instances and 10% of food-insecure instances (*p* < 0.001). All variables included are statistically significant predictors. Fit metrics for logistic regression indicate the predictive model performs adequately (Nagelkerke pseudo-*R*^2^ (coefficient of determination, measuring explained variation) = 0.165 and Cox and Snell pseudo-*R*^2^ = 0.100). Students who were living on their own were almost four times as likely to experience food insecurity with an odds ratio (OR) of 3.95. For graduate students (OR 2.883), those who were non-White (OR 2.27) and those who had either lost their job (OR 2.097) or were currently working (OR 1.808) had greater probability of being food insecure.

## 4. Discussion

The researchers hypothesized that employment and living situation changes related to the COVID-19 pandemic were associated with food insecurity among students at ISU. Multiple studies have shown an increased prevalence of food insecurity among college students and families during the pandemic compared to the rates preceding it [30,31,32]. However, the rates of early pandemic food insecurity were around 17% in the ISU students surveyed in April 2020, which were lower than previously recorded rates for ISU in the two preceding years (24–28%) as well as lower than national rates for college students (41%) [10,11,12]. Thus, the hypothesis was not supported although the factors associated with food security status were noticeably altered because of changed employment and living situation patterns.

The lower percentage for food insecurity may be due, in part, to the considerable number of students who moved home and had access to additional family resources. There was a significant shift in living situations following campus closure and more than half (53%) of the students in this study lived at home after COVID-19 restrictions. According to the Pew Research Center, about 22% of US adults knew someone who had to change their residency or they themselves had to change their residency due to COVID-19 [33]. Moves often occurred due to college dormitory closure, perception of unsafe communities, or the inability to afford housing [34]. In a similar pandemic study of students in Texas, researchers found that those living with parents or other relatives had lower odds of being food insecure compared to those living independently [34]. Another study showed students who moved in with family or otherwise received familial financial support had higher odds of reporting an improvement in food security status [30]. Similarly, a pre-pandemic study found that students living at home had lower rates of food insecurity [35].

Our study identified how the campus closure not only affected student living situations but also food procurement and preparation practices (Objective 1). Both students living on their own and food-insecure students expressed more frequent barriers to food access. Lack of time to prepare food is consistent with other research examining barriers [36]. Lack of time and inconvenient hours of operation may be related to the higher number of work hours for students living on their own and food-insecure students.

In this research, as in other studies, the frequency of student food insecurity is greater than the national average among the general population, yet students do not receive corresponding nutrition assistance [10,37]. A study conducted at Kansas State University found high food-insecurity rates, but only about 4% of students utilized any sort of nutrition assistance [37], which is similar to findings at ISU. The reasons students did not use food pantries included feeling that they did not need it, altruism, stigma, or the utilization of alternative coping mechanisms [37]. In the current study, among food-insecure students, almost 88% stated they did not utilize food assistance resources.

Students living with family consumed more home-prepared meals and may have had reduced responsibilities in food acquisition and preparation or possessed additional resources to prepare their own meals. These data suggest substantial familial support with food procurement. Similarly, food-secure students more frequently reported consuming homemade meals. Nationally, 62% of families decreased take-out/fast-food/already prepared meals and 73% increased consumption of home-cooked meals during the pandemic [31]. This study is consistent with a university study in Alabama, which found students living off campus and categorized as “very low food secure” had lower cooking self-efficacy scores and prepared meals at home less often [38]. Students living on their own and/or those with food insecurity may have less time to commit to meal preparation due to employment or other obligations, resulting in an increased reliance on take-out or fast food. The need to work longer hours in addition to keeping up with regular coursework could supplant the intent or desire to prepare home-cooked meals. Though potentially counterintuitive from a financial perspective, the tendency of food-insecure individuals to seek take-out or fast food more often than their food-secure peers is likely multifactorial and deserves additional scrutiny. For instance, the behavior might serve as a coping mechanism for greater stress levels.

Students living at home and food-secure students were more likely to buy shelf-stable foods routinely. Despite their shelf stability and affordability, less common categories for purchase were canned meats and dry beans, lentils, and chickpeas. This is a marked contrast from the overall trend in US consumer behavior in early March 2020 to purchase shelf-stable proteins. It may indicate an opportunity for the increased promotion of these products as convenient and nutritious food sources beyond the unique circumstances of the pandemic, particularly among college students living on their own or coping with food insecurity.

Students with food insecurity were more likely to stock up on some staple items. Typically, it is thought that food-insecure individuals are unable to purchase larger amounts of food at one time due to financial constraints but may buy less-expensive shelf-stable items that will last longer [2]. Given the unprecedented situation, it is possible that the combination of perceived food scarcity, the witnessing of panic buying, and the irrationality that can occur during an emergency resulted in additional anxiety in an already stressed population [39]. Food-insecure shoppers may also have stocked up to prepare home-cooked meals in lieu of eating out due to the pandemic.

Overall, few students met recommendations for fruit, vegetable, and fiber consumption (Table S1, consumption frequency of individual food items). However, fruit and vegetable intakes were greater in students living at home. Even under pre-pandemic conditions, college students generally did not consume enough of these foods [40]. Those who were food insecure reported lower health status, which was expected, but the intake for fruits and vegetables was not significantly different from that of food-secure students [13]. This finding is inconsistent with a study at a different university in the Midwest showing that food-insecure students had lower intakes of fruits, vegetables, and fiber compared to their food-secure peers [41].

Multiple differences between demographic and socioeconomic factors were found based on food security status (Objective 2). Factors associated with food insecurity included undergraduate status, non-White race, having a lower cooking self-efficacy score, receiving financial aid, being employed, having higher stress, living in the same situation as before the campus closure, and consuming more take-out or fast food. These findings are largely consistent with prior studies investigating college student food insecurity [10,13,21,38].

Compared to the national rate of 33% of Americans who reported high levels of psychological stress [3], the university sample result of 54% is a cause for concern. In general, Americans experiencing higher rates of stress were more likely to say that the pandemic had hurt them financially [3]. Thus, the high levels observed may have been a result of new circumstances. Food-insecure and non-White students reportedly experienced greater stress prior to the pandemic in other settings [42]. Food-insecure students had higher levels of binge drinking which may have been associated with additional stress.

The results of this study illuminated multiple factors associated with the risk of food insecurity under COVID-19 restricted conditions (Objective 3). Current living arrangement was the strongest predictor of food security status with students in this study. The greatest predictors of food insecurity among the students in Texas were current living arrangements and/or a loss or reduction in employment [34]. The ability, or need, to move home may have been influenced by work status with students in the current study. Students living at home were more likely either to have lost their job due to the pandemic shutdown or were not working for pay in the spring semester and students living on their own were more likely to be working over 11 h per week. Food-secure students may be more stable financially without the need to work for pay, while food-insecure students may work due to financial obligations. Significantly more food-insecure students also receive financial aid.

The logistic regression model demonstrates good predictive validity (*p* < 0.001), indicating that the selected predictors collectively provide meaningful indicators of student food security or insecurity in the era of COVID-19. Furthermore, the model correctly classifies a large percentage (83.5%) of observations. Given the relatively rare condition of food insecurity, the model correctly classifies 10.1% of such cases compared to 99.1% of cases of food security. Future models could use different combinations of variables with different degrees of predictive validity and subsequent analyses could use alternative cut-points other than the 0.5 value used in this analysis that would result in a greater ability to predict the rarer outcome of food insecurity.

Strengths of this study include having a large sample size and demographics comparable to the university overall. This study has several limitations. Although there is a large sample size, the data were collected from a convenience sample, which limited the generalizability of the findings. Additionally, the data represent a particular time period that is early relative to the COVID-19 pandemic and circumstances regarding financial aid and benefits may have altered since this study was performed. Questions were not asked specifically about changes due to COVID-19; rather, the inquiry was worded with respect to the four weeks prior to or at the time of survey participation.

## 5. Conclusions

The findings of this study reveal that food security status is largely predicted by whether students moved home to live with parents/guardians after campus closure due to the COVID-19 pandemic. Those living at home consumed more fruits and vegetables and had more home-prepared foods, experienced fewer barriers to food access and less severe stress, and had lower cooking self-efficacy. Food insecurity is prevalent on college campuses, with international and non-White students being the most impacted [11]. College student livelihoods have been largely altered by the COVID-19 pandemic. Implications of the pandemic also affected those who were the most food insecure due to pre-existing stressors, job loss, moving, and overall financial constraints.

These data support further research to assess effective programs that aim to reduce food insecurity on college campuses and its negative impact on students. For example, given that students experiencing food insecurity were more likely to have lower cooking self-efficacy scores, possibly due to arbitrary and subjective beliefs about the time and skill level required to prepare satisfying meals, interventions targeted at improving confidence could help to alleviate some of the distress related to food insecurity. This research highlights the characteristics of students who may be at higher risk and, as such, could receive proactive outreach. Additionally, since this study elucidates a specific time during the pandemic, continued assessment of college student behaviors and livelihoods as affected by the pandemic is warranted.

## Figures and Tables

**Table 1:** Demographic characteristics of Midwestern university students by residence and food security status post-COVID-19 campus shift to online instruction (%; *n* = 1434).

		Residence			Food Security Status		
Characteristics	Total (%)	At Home 53% (759)	On Own 47% (675)	*p*	Food Secure 83% (1184)	Food Insecure 17% (250)	*p*
Age in Years (mean ± SD)	21.4 ± 2.7	20.0 ± 1.6	22.0 ± 2.9	<0.001	21.3 ± 2.7	21.8 ± 2.8	0.011
		←%→			←%→		
Gender							
Male	38.8	34.8 _a_	43.4 _b_	0.001	39.0	38.0	0.764
Female	61.2	65.2 _a_	56.6 _b_		61.0	62.0	
Race				<0.001			<0.001
White	81.9	88.4 _a_	74.5 _b_		84.3 _a_	70.4 _b_	
Other Race	18.1	11.6 _a_	25.5 _b_		15.7 _a_	29.6 _b_	
Undergraduate/Graduate				<0.001			0.490
Undergraduate	82.1	97.0 _a_	65.3 _b_		81.8	83.6	
Graduate	17.9	3.0 _a_	34.7 _b_		18.2	16.4	
Residency status							
Iowa student	55.9	61.5 _a_	49.5 _b_	<0.001	55.9 _a_	55.6 _a_	0.001
US—other states	35.1	37.7 _a_	32.1 _b_		36.2 _a_	29.6 _b_	
International	9.1	0.8 _a_	18.4 _b_		7.8 _a_	14.8 _b_	
Marital status							
Single/divorced/widowed	83.4	97.2 _a_	67.8 _b_	<0.001	85.0 _a_	75.8 _b_	<0.001
Married or cohabitating	16.6	2.8 _a_	32.2 _b_		15.0 _a_	24.2 _b_	
Receive financial aid				0.064			0.004
Yes	66.6	68.7	64.1		64.9 _a_	74.3 _b_	
No	33.4	31.3	35.9		35.1 _a_	25.7 _b_	
Self-reported health status				0.174			<0.001
Poor-Fair	12.3	11.2	13.6		10.3 _a_	22.3 _b_	
Good	42.0	41.0	43.0		41.8 _a_	42.9 _a_	
Very Good-Excellent	45.7	47.8	43.4		48.0 _a_	34.7 _b_	
Body Mass Index (BMI)				0.470			0.002
Underweight	3.6	4.0	3.1		3.7 _a_	3.2 _a_	
Normal	57.8	58.4	57.2		59.1 _a_	51.8 _a_	
Overweight	26.1	26.3	26.0		26.4 _a_	25.1 _b_	
Obese	12.4	11.3	13.7		10.9 _a_	19.8 _b_	
Daily fruit/vegetable servings				0.001			0.711
<5 per day	86.5	83.5 _a_	89.8 _b_		86.3	87.2	
5 or more per day	13.5	16.5 _a_	10.2 _b_		13.7	12.8	
Daily fiber servings				0.313			0.094
<20 g per day	66.7	67.9	65.3		65.7	71.2	
20+ grams per day	33.3	32.1	34.7		34.3	28.8	

Chi-square test for independence of variables was assessed. Same subscript letters (_a,b_) indicate column proportions that are not significantly different from each other.

**Table 2:** COVID-19-related behaviors in previous four weeks of Midwestern university students by residence and food security status (*n* = 1434).

		Residence			Food Security Status		
	Total (%)	At Home 53% (759)	On Own 47% (675)	*p*	Food Secure 83% (1184)	Food Insecure 17% (250)	*p*
Behaviors		←%→			←%→		
Housing change in March 2020				<0.001			<0.001
Did not change residence	50.8	16.1 _a_	89.9 _b_		47.8 _a_	65.2 _b_	
Moved due to COVID-19	30.7	54.0 _a_	4.4 _b_		32.5 _a_	22.0 _b_	
Moved to be closer to family	15.9	28.5 _a_	1.8 _b_		17.1 _a_	10.0 _b_	
Moved for other reason	2.6	1.4 _a_	3.9 _b_		2.5 _a_	2.8 _a_	
Pre-COVID residence				<0.001			0.004
Parent/Guardian Home	9.0	16.6 _a_	0.4 _b_		9.9 _a_	4.8 _b_	
Off campus housing	59.6	40.6 _a_	81.0 _b_		57.9 _a_	68.0 _b_	
On campus housing	31.4	42.8 _a_	18.5 _b_		32.3 _a_	27.2 _a_	
Current residence				N/A			<0.001
Parent/Guardian Home	53.0	100.0	---		58.0 _a_	29.6 _b_	
Off campus housing	36.8	---	78.3		33.7 _a_	51.6 _b_	
On campus housing	7.7	---	16.5		6.4 _a_	14.0 _b_	
Temporary housing	2.4	---	5.0		1.9 _a_	4.8 _b_	
Employment status				<0.001			0.001
Not working for pay	26.9	37.2 _a_	15.2 _b_		29.2 _a_	16.1 _b_	
Lost job in past 4 weeks	20.6	24.1 _a_	16.5 _b_		19.6 _a_	25.0 _a_	
Working 1–10 h/week	16.0	16.8 _a_	15.2 _a_		15.1 _a_	20.2 _b_	
Working 11–20 h/week	19.6	13.1 _a_	27.0 _b_		19.2 _a_	21.4 _a_	
Working 21+ h/week	16.9	8.8 _a_	26.1 _b_		16.8 _a_	17.3 _a_	
Self-isolation practice				<0.001			0.091
All of the time	20.5	27.8 _a_	12.2 _b_		21.3	16.5	
Most of the time	54.3	48.8 _a_	60.5 _b_		54.4	53.8	
Some or none of the time	25.2	23.4 _a_	27.3 _a_		24.3	29.7	
Stress				0.035			<0.001
Less than average or none	13.1	11.9 _a_	14.6 _a_		13.6 _a_	10.8 _a_	
Average stress	32.7	33.1 _a_	32.3 _a_		34.1 _a_	26.4 _b_	
More than average stress	45.0	47.5 _a_	42.3 _b_		44.8 _a_	46.0 _a_	
Tremendous stress	9.1	7.5 _a_	10.9 _b_		7.5 _a_	16.8 _b_	
Alcohol—5 or more per time				<0.001			<0.001
Do not drink alcohol	24.8	32.0 _a_	16.5 _b_		25.2 _a_	22.8 _a_	
None	46.9	47.2 _a_	46.6 _a_		48.9 _a_	37.8 _b_	
1 time	10.4	8.3 _a_	12.8 _b_		10.0 _a_	12.6 _a_	
2–3 times	11.1	8.3 _a_	14.3 _b_		10.6 _a_	13.4 _a_	
4 or more times	6.8	4.1 _a_	9.8 _b_		5.4 _a_	13.4 _b_	

N/A = not applicable. Same subscript letters (_a,b_) indicate column proportions that are not significantly different.

**Table 3:** Food acquisition and preparation characteristics of food of Midwestern university students by residence and food security status post-COVID-19 campus shift to online instruction (*n* = 1434).

		Residence			Food Security Status		
	Total (%)	At Home 53% (759)	On Own 47% (675)	*p*	Food Secure 83% (1184)	Food Insecure 17% (250)	*p*
Food acquisition and preparation		←%→			←%→		
Main food shopper				<0.001			<0.001
Self buys most food	36.1	8.8 _a_	66.7 _b_		32.3 _a_	53.6 _b_	
Self and another 50/50	21.2	15.8 _a_	27.3 _b_		19.6 _a_	28.8 _b_	
Someone else buys	42.7	75.4 _a_	6.1 _b_		48.1 _a_	17.6 _b_	
Weekly shopping frequency				<0.001			<0.001
Less than 1 time	23.3	16.0 _a_	31.4 _b_		21.4 _a_	32.3 _b_	
1 time	52.0	52.9 _a_	50.9 _a_		52.9 _a_	47.6 _a_	
2 or more times	24.8	31.0 _a_	17.7 _b_		25.7 _a_	20.2 _a_	
Main meal preparer							<0.001
Self	37.5	16.0 _a_	61.5 _b_		34.5 _a_	51.8 _b_	
Self and another 50/50	32.9	34.4 _a_	31.3 _a_	<0.001	33.4 _a_	30.9 _a_	
Someone else prepares	29.6	49.5 _a_	7.3 _b_		32.2 _a_	17.3 _b_	
Cooking Self-Efficacy Score (mean ± SD)	23.2 ± 4.3	22.8 ± 4.4	23.7 ± 4.1	<0.001	23.3 ± 4.1	22.2 ± 4.3	0.006
Barriers to food access							
No time to shop for food				<0.001			<0.001
Very Often/Often	6.3	3.6 _a_	9.4 _b_		4.1 _a_	16.9 _b_	
Sometimes	15.3	11.7 _a_	19.3 _b_		12.1 _a_	30.5 _b_	
Rarely/Never	78.4	84.7 _a_	71.3 _b_		83.8 _a_	52.6 _b_	
No time to prepare food				<0.001			<0.001
Very Often/Often	8.9	5.1 _a_	13.1 _b_		5.8 _b_	23.2 _b_	
Sometimes	19.8	15.7 _a_	24.3 _b_		17.8 _b_	29.2 _b_	
Rarely/Never	71.3	79.2 _a_	62.6 _b_		76.4 _b_	47.6 _b_	
No facilities to cook/store food				0.005			<0.001
Very Often/Often	1.2	0.4 _a_	2.1 _b_		0.3 _a_	5.2 _b_	
Sometimes	5.4	4.6 _a_	6.2 _a_		3.7 _a_	13.3 _b_	
Rarely/Never	93.4	95.0 _a_	91.7 _b_		95.9 _a_	81.5 _b_	
Lack of transportation				<0.001			<0.001
Very Often/Often	3.8	1.7 _a_	6.2 _b_		2.1 _a_	12.0 _b_	
Sometimes	5.2	4.2 _a_	6.4 _a_		4.5 _a_	8.8 _b_	
Rarely/Never	90.9	94.0 _a_	87.4 _b_		93.4 _a_	79.1 _b_	
Cost of food				<0.001			<0.001
Very Often/Often	8.9	4.5 _a_	13.8 _b_		3.1 _a_	36.0 _b_	
Sometimes	20.2	14.9 _a_	26.1 _b_		15.1 _a_	44.4 _b_	
Rarely/Never	70.9	80.6 _a_	60.1 _b_		81.8 _a_	19.6 _b_	
Location of food outlets not easy to get to				0.024			<0.001
Very Often/Often	4.9	3.6 _a_	6.4 _b_		2.5 _a_	16.0 _b_	
Sometimes	9.2	8.5 _a_	10.1 _a_		8.0 _a_	15.2 _b_	
Rarely/Never	85.9	87.9 _a_	83.6 _b_		89.5 _a_	68.8 _b_	
Hours of operation of food outlets				<0.001			<0.001
Very Often/Often	7.8	4.2 _a_	11.7 _b_		4.2 _a_	24.5 _b_	
Sometimes	17.2	15.2 _a_	19.4 _b_		16.5 _a_	20.5 _a_	
Rarely/Never	75.1	80.6 _a_	68.8 _b_		79.3 _a_	55.0 _b_	
Lack of availability of cultural or ethnic foods				<0.001			<0.001
Very Often/Often	7.8	4.4 _a_	11.6 _b_		5.6 _a_	18.0 _b_	
Sometimes	11.1	9.4 _a_	13.0 _b_		9.5 _a_	18.4 _b_	
Rarely/Never	81.1	86.2 _a_	75.4 _b_		84.9 _a_	63.6 _b_	
Lack of foods for dietary needs				0.002			<0.001
Very Often/Often	3.7	2.4 _a_	5.2 _b_		1.9 _a_	12.4 _b_	
Sometimes	9.6	8.1 _a_	11.3 _b_		7.7 _a_	18.4 _b_	
Rarely/Never	86.7	89.5 _a_	83.5 _b_		90.4 _a_	69.2 _b_	
Utilization of food assistance resources							
Used food assistance or food pantry				0.025			<0.001
Yes	3.8	2.8 _a_	5.1 _b_		2.0 _a_	12.4 _b_	
No	96.2	97.2 _a_	94.9 _b_		98.0 _a_	87.6 _b_	

Same subscript letters (_a,b_) indicate column proportions that are not significantly different from each other.

**Table 4:** Main meal source and pandemic stock up purchase behavior of Midwestern university students by residence and food security status post-COVID-19 campus shift to online instruction (*n* = 1434).

		Residence			Food Security Status		
	Total	At Home 53% (759)	On Own 47% (675)	*p*	Food Secure 83% (1184)	Food Insecure 17% (250)	*p*
Main Meal Source		←%→			←%→		
Homemade food				0.013			<0.001
Never	1.5	1.1 _a_	2.1 _a_		1.2 _a_	3.2 _b_	
1–3 days per month	4.1	3.2 _a_	5.2 _a_		3.6 _a_	6.4 _b_	
1–2 days per week	9.7	8.3 _a_	11.3 _a_		8.1 _a_	17.2 _b_	
3 or more days per week	84.7	87.5 _a_	81.5 _b_		87.1 _a_	73.2 _b_	
Microwave/frozen meals				0.027			0.002
Never	17.4	15.4 _a_	19.7 _b_		17.3 _a_	17.7 _a_	
1–3 days/month	30.8	29.7 _a_	32.1 _a_		31.9 _a_	25.7 _a_	
1–2 days/week	33.1	36.2 _a_	29.6 _b_		33.8 _a_	29.7 _a_	
3 or + days/week	18.7	18.8 _a_	18.7 _a_		17.0 _a_	26.9 _b_	
Take-out food, restaurant				0.175			0.003
Never	14.5	13.2	16.0		14.4 _a_	14.9 _a_	
1–3 days per month	47.9	46.8	49.2		47.0 _a_	52.4 _a_	
1–2 days per week	33.7	35.7	31.5		35.4 _a_	25.8 _b_	
3 or more days per week	3.8	4.3	3.3		3.2 _a_	6.9 _b_	
Fast food drive through				0.004			<0.001
Never	30.2	29.2 _a_	31.3 _a_		31.9 _a_	22.0 _b_	
1–3 days per month	46.2	50.1 _a_	41.8 _b_		46.4 _a_	45.2 _a_	
1–2 days per week	20.8	18.7 _a_	23.1 _b_		19.8 _a_	25.2 _a_	
3 or more days per week	2.9	2.0 _a_	3.9 _b_		1.9 _a_	7.6 _b_	
Campus outlet take-out				0.003			<0.001
Never	86.3	89.0 _a_	83.3 _b_		88.2 _a_	77.6 _b_	
1–3 days per month	7.5	5.4 _a_	9.8 _b_		6.8 _a_	10.8 _b_	
1–2 days per week	2.5	2.8 _a_	2.2 _a_		2.0 _a_	4.8 _b_	
3 or more days per week	3.6	2.8 _a_	4.6 _b_		3.0 _a_	6.8 _b_	
Pandemic stock up purchases							
Pasta, spaghetti, and noodles				<0.001			0.002
Buy routinely	61.9	68.3 _a_	54.6 _b_		63.5 _a_	53.8 _b_	
Bought to stock up	22.9	22.1 _a_	23.7 _a_		22.7 _a_	23.5 _a_	
First time purchase	3.4	3.0 _a_	3.7 _a_		2.9 _a_	5.7 _b_	
Did not buy	11.9	6.6 _a_	17.9 _b_		10.9 _a_	17.0 _b_	
Rice or potatoes				0.001			<0.001
Buy routinely	67.9	72.4 _a_	62.8 _b_		70.3 _a_	56.5 _b_	
Bought to stock up	18.5	16.9 _a_	20.2 _a_		17.2 _a_	24.6 _b_	
First time purchase	3.1	2.6 _a_	3.7 _a_		2.5 _a_	6.5 _b_	
Did not buy	10.5	8.1 _a_	13.2 _b_		10.1 _b_	12.5 _b_	
Dry beans, lentils, and chickpeas				0.003			0.009
Buy routinely	20.5	24.3 _a_	16.2 _b_		21.4 _a_	16.1 _a_	
Bought to stock up	8.8	8.2 _a_	9.5 _a_		7.8 _a_	13.7 _b_	
First time purchase	6.1	5.8 _a_	6.4 _a_		5.9 _a_	7.3 _a_	
Did not buy	64.6	61.7 _a_	67.9 _b_		65.0 _a_	62.9 _a_	
Canned beans or legumes				<0.001			<0.001
Buy routinely	35.9	41.8 _a_	29.4 _b_		38.5 _a_	23.8 _b_	
Bought to stock up	14.2	14.0 _a_	14.3 _a_		13.9 _a_	15.3 _a_	
First time purchase	6.6	6.1 _a_	7.2 _a_		5.6 _a_	11.3 _b_	
Did not buy	43.3	38.1 _a_	49.2 _b_		42.0 _a_	49.6 _b_	
Frozen fruits or vegetables				<0.001			<0.001
Buy routinely	57.4	63.5 _a_	50.4 _b_		59.6 _a_	46.6 _b_	
Bought to stock up	16.0	15.5 _a_	16.6 _a_		16.0 _a_	16.1 _a_	
First time purchase	5.3	5.2 _a_	5.5 _a_		4.1 _a_	11.2 _b_	
Did not buy	21.3	15.9 _a_	27.5 _b_		20.3 _a_	26.1 _b_	
Canned vegetables or fruit				<0.001			0.010
Buy routinely	41.8	50.9 _a_	31.6 _b_		43.5 _a_	33.5 _b_	
Bought to stock up	16.7	18.6 _a_	14.6 _b_		16.0 _a_	20.2 _a_	
First time purchase	6.7	5.7 _a_	7.7 _a_		6.0 _a_	9.7 _b_	
Did not buy	34.8	24.8 _a_	46.1 _b_		34.4 _a_	36.7 _a_	
Canned meat (tuna, SPAM)				<0.001			0.108
Buy routinely	22.3	26.5 _a_	17.6 _b_		23.3	17.6	
Bought to stock up	10.4	11.5 _a_	9.1 _a_		10.1	11.6	
First time purchase	5.1	4.2 _a_	6.1 _a_		4.7	7.2	
Did not buy	62.3	57.8 _a_	67.3 _b_		62.0	63.6	
Snacks (chips, crackers, and cookies)				<0.001			<0.001
Buy routinely	65.1	73.2 _a_	56.0 _b_		68.5 _a_	49.0 _b_	
Bought to stock up	12.9	12.5 _a_	13.3 _a_		11.7 _a_	18.5 _b_	
First time purchase	4.4	2.9 _a_	6.0 _b_		3.7 _a_	7.2 _b_	
Did not buy	17.7	11.4 _a_	24.7 _b_		16.1 _a_	25.3 _b_	

Same subscript letters (_a,b_) indicate column proportions that are not significantly different from each other.

**Table 5:** Logistic regression model of predictors of food insecurity among Midwestern university students post-COVID closure of campus in March 2020 (*n* = 1434).

			95% Confidence Interval		
	Beta (SE)	*p*	Lower	Odds Ratio	Upper
Cooking self-efficacy	−0.058 (0.017)	0.001	0.912	0.944	0.976
Living at parents or on own (1)	1.374 (0.167)	<0.001	2.848	3.951	5.481
Graduate student status (1)	1.059 (0.223)	<0.001	1.863	2.883	4.463
Non-White race (1)	0.820 (0.180)	<0.001	1.595	2.270	3.229
Stress	0.424 (0.091)	<0.001	1.277	1.528	1.827
Employment		0.004			
Lost job due to COVID-19	0.741 (0.233)	00.001	1.328	2.097	3.312
Currently working	0.592 (0.209)	0.005	1.200	1.808	2.724
Constant	−3.641 (0.561)	<0.001		0.026	
Percent correct	Food Secure		99.1	Overall	83.5
	Food Insecure		10.1		
Model significance	*p* < 0.001				

## Data Availability

The data presented in this study are available upon request from the corresponding author. The data are not publicly available due to privacy reasons.

## Supplementary Materials

The following are available online at https://www.mdpi.com/article/10.3390/nu13061932/s1, Table S1: Consumption frequency of individual food items of Midwestern university students by residence and food security status post-COVID-19 campus shift to online instruction, Table S2: Cooking self-efficacy of Midwestern university students by residence and food security status post-COVID-19 campus shift to online instruction.
