# Supplementary material for: Effects of COVID-19 on University Student Food Security
Source: Nutrients. 2021 Jun 4;13(6):1932. doi: 10.3390/nu13061932 (PMC8229979; doi:10.3390/nu13061932)
Supplement: Supplementary file 1 [file nutrients-13-01932-s001.zip › nutrients-1206523-supplementary.pdf]

nutrients

MDPI

Table S1. Consumption frequency of individual food items of Midwestern university students by residence and food security status post-COVID-19 campus shift to online instruction (n=1434).

		Residence			Food Security Status		
	Total (%)	At Home 53% (759)	On Own 47% (675)	p	Food Secure 83% (1,184)	Food Insecure 17% (250)	p
		←—%	—→		←—%	—→	
Fruit juice				<.001			.626
< 1 time per week	47.4	41.8_a	53.6_b		48.1	43.6	
Once per week	17.7	19.0_a	16.3_a		17.1	20.4	
2-3 times per week	18.8	20.6_a	16.9_a		18.6	20.0	
4-6 times per week	7.9	9.5_a	6.1_b		7.8	8.4	
1 or more times per day	8.2	9.2_a	7.1_a		8.4	7.6	
Whole fruit				<.001			<.001
< 1 time per week	8.9	6.6_a	11.4_b		7.1_a	17.2_b	
Once per week	12.9	9.9_a	16.3_b		11.8_a	18.0_b	
2-3 times per week	23.8	21.1_a	27.0_b		23.1_a	27.2_a	
4-6 times per week	22.6	26.1_a	18.7_b		23.6_a	18.0_a	
1 or more times per day	31.8	36.4_a	26.7_b		34.4_a	19.6_b	
Vegetable juice				.837			.001
< 1 time per week	88.9	88.1	89.8		90.4_a	82.0_b	
Once per week	5.2	5.5	4.7		4.7_a	7.2_a	
2-3 times per week	3.3	3.7	2.8		2.9_a	5.2_a	
4-6 times per week	1.0	0.9	1.0		0.7_a	2.4_b	
1 or more times per day	1.7	1.7	1.6		1.4_a	3.2_b	
Potatoes				<.001			.588
< 1 time per week	10.0	5.5_a	15.0_b		9.6	11.6	
Once per week	23.2	20.4_a	26.4_b		24.0	19.6	
2-3 times per week	41.2	42.0_a	40.3_a		40.8	43.2	
4-6 times per week	20.2	24.8_a	15.0_b		20.2	20.0	
1 or more times per day	5.4	7.2_a	3.4_b		5.4	5.6	
Green salad				<.001			.277
< 1 time per week	28.3	23.3_a	33.9_b		27.4	32.8	
Once per week	25.8	25.4_a	26.2_a		25.5	27.2	
2-3 times per week	26.6	28.6_a	24.4_a		27.1	24.4	
4-6 times per week	11.2	13.0_a	9.2_b		11.7	8.8	
1 or more times per day	8.0	9.6_a	6.2_b		8.3	6.8	
Vegetable stews				<.001			.290
< 1 time per week	57.3	57.0_a	57.6_a		57.4	56.8	
Once per week	26.5	30.0_a	22.5_b		27.2	23.2	
2-3 times per week	11.7	9.6_a	14.1_b		11.3	13.6	
4-6 times per week	2.7	2.4_a	3.1_a		2.5	3.6	
1 or more times per day	1.7	0.9_a	2.7_b		1.5	2.8	
Other vegetables				.001			<.001
< 1 time per week	6.1	4.7_a	7.6_b		5.2_a	10.4_b	
Once per week	10.5	8.0_a	13.3_b		9.1_a	17.2_b	
2-3 times per week	29.8	31.0_a	28.4_a		29.5_a	31.2_a	
4-6 times per week	22.0	23.7_a	20.0_a		22.8_a	18.0_a	
1 or more times per day	31.7	32.5_a	30.7_a		33.4_a	23.2_b	
Fiber cereals				.163			.228
< 1 time per week	63.7	62.5	65.0		64.8	58.4	
Once per week	13.5	14.0	12.9		13.3	14.0	
2-3 times per week	13.6	14.4	12.7		13.3	15.2	
4-6 times per week	5.2	4.3	6.2		4.7	7.6	
1 or more times per day	4.0	4.9	3.1		3.9	4.8	
Beans, peas, lentils, etc.				.271			.690

< 1 time per week	38.7	38.5	39.0		38.1	41.6	
Once per week	25.9	26.7	24.9		26.5	22.8	
2-3 times per week	20.6	21.6	19.6		20.4	21.6	
4-6 times per week	8.2	7.8	8.6		8.4	7.2	
1 or more times per day	6.6	5.4	8.0		6.6	6.8	
Dark breads				.124			.378
< 1 time per week	43.7	44.0	43.3		43.2	46.0	
Once per week	15.9	17.7	13.9		16.4	13.6	
2-3 times per week	20.8	20.4	21.2		20.2	23.6	
4-6 times per week	11.7	11.3	12.1		11.9	10.8	
1 or more times per day	7.9	6.6	9.5		8.4	6.0	

Same subscript letters (a, b) indicate column proportions that are not significantly different from each other.

Table S2. Cooking self-efficacy of Midwestern university students by residence and food security status post-COVID-19 campus shift to online instruction (*n*=1434).

		Residence			Food Security Status		
	Total (%)	At Home 53% (759)	On Own 47% (675)	*p*	Food Secure 83% (1184)	Food Insecure 17% (250)	*p*
		←%→			←%→		
Follow recipe direction				.166			<.001
Not at all confident	0.6	0.5	0.7		0.4$_a$	1.6$_b$	
A little confident	2.1	1.5	2.8		1.4$_a$	5.6$_b$	
Somewhat confident	15.0	16.2	13.6		14.8$_a$	16.0$_a$	
Very confident	82.3	81.8	82.8		83.4$_a$	76.8$_b$	
Prepare a meal				<.001			.216
Not at all confident	1.4	2.0$_a$	0.7$_b$		1.4	1.6	
A little confident	5.5	6.7$_a$	4.1$_b$		5.0	8.0	
Somewhat confident	18.4	23.1$_a$	13.2$_b$		18.9	16.0	
Very confident	74.7	68.2$_a$	81.9$_b$		74.7	74.4	
Make a lettuce and chopped vegetable salad				.422			<.001
Not at all confident	2.9	2.4	3.4		2.3$_a$	5.6$_b$	
A little confident	6.7	6.1	7.4		5.6$_a$	12.0$_b$	
Somewhat confident	16.2	6.9	15.4		16.3$_a$	15.6$_a$	
Very confident	74.2	74.7	73.8		75.8$_a$	66.8$_b$	
Prepare a mixture such as a stir-fry				<.001			.007
Not at all confident	7.7	10.3$_a$	4.9$_b$		7.4$_a$	9.2$_a$	
A little confident	16.2	18.2$_a$	13.9$_b$		15.7$_a$	18.4$_a$	
Somewhat confident	25.1	27.2$_a$	22.7$_b$		23.8$_a$	31.2$_b$	
Very confident	51.0	44.3$_a$	58.5$_b$		53.1$_a$	41.2$_b$	
Prepare a mixture such as a casserole or 'hotdish'				.008			.001
Not at all confident	9.8	10.7$_a$	8.7$_a$		8.5$_a$	16.0$_b$	
A little confident	18.4	20.2$_a$	16.3$_a$		18.5$_a$	17.6$_a$	
Somewhat confident	24.2	25.7$_a$	22.5$_a$		23.7$_a$	26.8$_a$	
Very confident	47.7	43.4^a	52.4$_b$		49.4$_a$	39.6$_b$	
Prepare baked goods (such as bread, cake, cookies, or muffins) from scratch				.016			.008
Not at all confident	11.2	9.9$_a$	12.6$_a$		10.0$_a$	16.8$_b$	
A little confident	16.8	16.4$_a$	17.3$_a$		16.5$_a$	18.4$_a$	
Somewhat confident	22.3	20.2$_a$	24.6$_b$		23.1$_a$	18.4$_a$	
Very confident	49.8	53.6$_a$	45.5$_b$		50.5$_a$	46.4$_a$	
Create a hot meal from scratch without needing to follow a recipe				<.001			.300
Not at all confident	19.8	25.6$_a$	13.3$_b$		19.9	19.2	
A little confident	22.2	26.1$_a$	17.8$_b$		22.8	19.2	
Somewhat confident	21.4	21.4$_a$	21.5$_a$		21.7	20.0	
Very confident	36.6	26.9$_a$	47.4$_b$		35.5	41.6	

Same subscript letters (a, b) indicate column proportions that are not significantly different from each other.
